# Polycrystalline Ni nanotubes under compression: a molecular dynamics study

**DOI:** 10.1038/s41598-020-76276-y

**Published:** 2020-12-03

**Authors:** J. Rojas-Nunez, S. E. Baltazar, R. I. Gonzalez, E. M. Bringa, S. Allende, M. Kiwi, F. J. Valencia

**Affiliations:** 1 Departamento de Física, Universidad de Santiago de Chile, USACH, Av. Ecuador, 3493 Santiago, Chile; 2 CEDENNA, Universidad de Santiago de Chile, USACH, Av. Ecuador, 3493 Santiago, Chile; 3 Centro de Nanotecnología Aplicada, Facultad de Ciencias, Universidad Mayor, Providencia, Chile; 4 CONICET and Facultad de Ingeniería, Universidad de Mendoza, 5500 Mendoza, Argentina; 5 Centro de Investigación DAiTA Lab, Facultad de Estudios Interdisciplinarios, Universidad Mayor, Santiago, Chile; 6 Departamento de Física, Facultad de Ciencias, Universidad de Chile, Santiago, Chile

**Keywords:** Nanowires, Mechanical properties

## Abstract

Mechanical properties of nanomaterials, such as nanowires and nanotubes, are an important feature for the design of novel electromechanical nano-architectures. Since grain boundary structures and surface modifications can be used as a route to modify nanostructured materials, it is of interest to understand how they affect material strength and plasticity. We report large-scale atomistic simulations to determine the mechanical response of nickel nanowires and nanotubes subject to uniaxial compression. Our results suggest that the incorporation of nanocrystalline structure allows completely flexible deformation, in sharp contrast with single crystals. While crystalline structures at high compression are dominated by dislocation pinning and the multiplication of highly localized shear regions, in nanocrystalline systems the dislocation distribution is significantly more homogeneous. Therefore, for large compressions (large strains) coiling instead of bulging is the dominant deformation mode. Additionally, it is observed that nanotubes with only 70% of the nanowire mass but of the same diameter, exhibit similar mechanical behavior up to 0.3 strain. Our results are useful for the design of new flexible and light-weight metamaterials, when highly deformable struts are required.

## Introduction

The development of lightweight hierarchical metallic nanostructures constitutes an alternative to create materials with novel and unexpected mechanical properties, a subject that recently has attracted much attention and has been extensively studied^1,2^. Usually, these structures correspond to a collection of mostly nanocrystalline (nc) nanotubes (NTs) or nanowires (NWs), self-assembled as building blocks and suitably arranged to ensure, for a given deformation, a cooperative response of all constituents^3–6^. Thus, the mechanical characteristics are dependent on the nanostructure design, and on the properties of the nano-objects used as building blocks^7^. Extensive research has been conducted on the mechanical properties of metallic crystalline (c-NWs) and nanocrystalline nanowires (nc-NWs)^8–13^. However, the hollow counterpart has not received much attention, probably due to the lack of experimental data on these kind of materials. Nowadays, the development of atomic layer deposition techniques and the Kirkendall effect allow the successful synthesis of nanocrystalline nanotubes with an adjustable combination of geometrical parameters^14–16^, and a nanocrystalline texture close to 5 nm grain size^17^. It is worth noticing that NTs are a lightweight version of NWs, but with a larger surface to volume ratio and smaller specific density, features that make them excellent candidates for their use in catalysis, energy devices, and the design of novel nanostructures^18^. The use of NTs and NWs for nanostructured devices requires an understanding of how the filaments respond under tensile and compressive deformation. Molecular dynamics simulations have shown that single crystal NT mechanical properties can be tailored by adjusting both inner and outer surfaces^19–21^.

However, simulations of nc-NTs have not received the same attention as simulations of c-NTs , even though they are an excellent approximation to the typically obtained experimental conditions^16^. A detailed study of Ni nc-NTs under tension was carried out by Rojas-Nunez et al.^17^ revealing that nc-NTs in the elastic regime, with similar yield strength and a mass reduction close to 60%, behave similarly to nc-NWs. They have just a slightly different fracture point. Finite element models under compression computed buckling of thin-walled NTs, but the model did not include complex defects, such as dislocations or grain boundaries^22^. On the other hand, molecular dynamics simulations show that thin crystalline face centered cubic (fcc) NWs deform by stacking faults and twins driven by surface tensile stress, assisted by partial dislocations^10^. Monk and Farkas^23^ showed the asymmetric behavior under tension-compression of Ni nc-NWs of 10 nm grain size, and concluded that plasticity in relatively small diameter NWs is mediated by grain boundary sliding, rather than by dislocation activity. Modeling nc-NWs and nc-NTs under experimental conditions implies grain sizes where grain boundary activity cannot be safely ignored. Additionally, the large specific surface areas of NTs allows a richer behavior of grain boundary and dislocation motion. Both factors could lead to materials with unexpected mechanical properties. To treat all these issues we implemented molecular dynamics simulations to investigate the effect of mechanical compression on nanocrystalline and crystalline NTs and NWs. We studied the dominant mechanisms in the plastic regime for large compressive deformations, and compared the difference between nc-NTs and nc-NWs with the respective pristine counterparts. In addition, the way these systems recover from different deformation scenarios is discussed.

## Results

### Mechanical compression of Ni nanotubes and nanowires

To compare the plasticity mechanisms of crystalline and nanocrystalline materials we carried out a comparison of the same diameter (22 nm) nanotubes and nanowires. The aspect ratio, defined as the ratio between NT length and thickness, was set to 4.8 for all samples. The relevance of the aspect ratio in the ductile to brittle fracture of nanocrystalline nanowires was discussed by Wu et al.^24^; we adopted the aspect ratio of 4.8 to reduce possible finite size effects due to the characteristic length scale of the plastic deformation sources, such as dislocations, and fracture or shear localization zones^25^.

**Figure 1:**
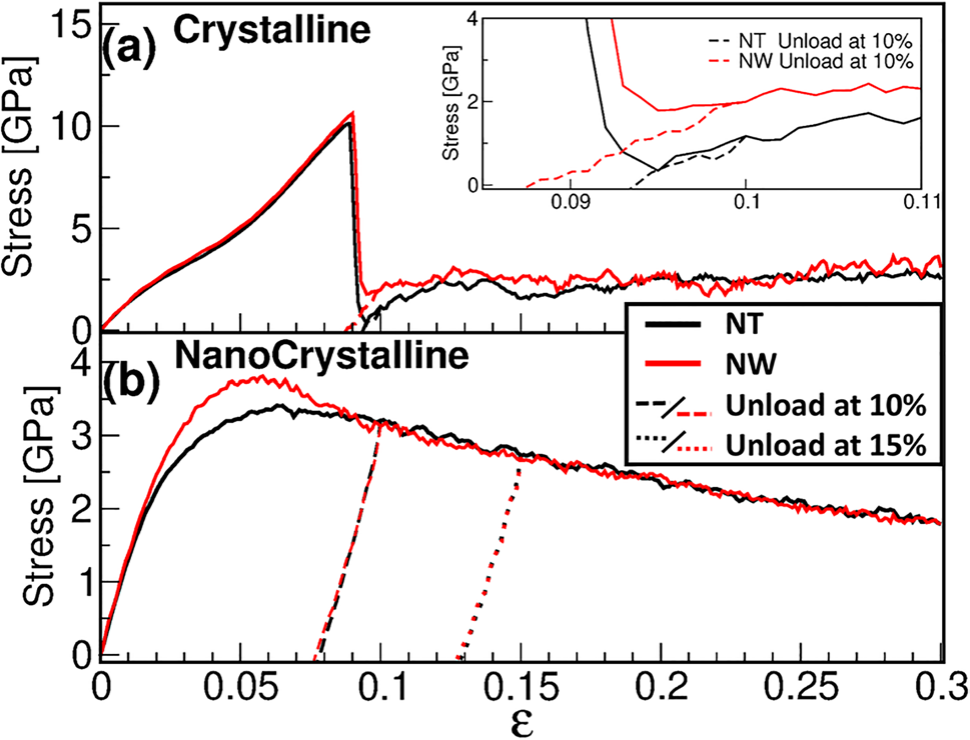
Compression stress-strain plots for (**a**) crystalline NT and NW with 22nm external diameter; (**b**) nanocrystalline NT and NW with 22 nm external diameter and 5 nm wall thickness. Dashed lines correspond to recovery during unloading to zero stress.

Uniaxial compression is applied to both crystalline and nanocrystalline NTs and NWs until a strain of 0.3 is reached. Fig. 1a shows the behavior of c-NTs and c-NWs, whose characteristic elastic regime extends up to strains of 0.09, and includes non-linear elasticity. The plasticity starts with a significant stress drop right after the ultimate strength for both structures is achieved, with a stress reduction of nearly 97% for NTs, and 84% for NWs. This abrupt fall is due to multiple slips in the preferential planes^23^ along the NT (and NW) diameter^10^, assisted by partial dislocations across the NT/NW that leave stacking faults behind. The DXA algorithm from OVITO provides identification of different types of dislocations, including fcc crystals full, Shockley, stair-rod, Hirth, and Frank dislocations. OVITO does not provide information on dislocation junctions, but stair-rod, Hirth, and Frank dislocations are sessile dislocations which typically appear as the result of dislocation junctions^26^. The c-NW stress drop for $\epsilon$ = 0.1 is caused by the nucleation and propagation of partial dislocations, usually associated with softening. However, the large number of partials rapidly leads to multiple dislocation junctions, identified by OVITO as a large number of sessile stair-rod dislocations which lead to hardening. Both effects produce a nearly constant stress flow at 2.5 GPa, for NWs and NTs. Fig. S1 illustrates this, showing the dislocation network for a c-NW.

On the other hand, both NTs and NWs with nanocrystalline structures show a completely different macroscopic behavior. Figure 1b reveals a smooth transition from elastic to plastic regimes, as expected from previous nc simulations^17^. At the nanoscale, metallic single crystal deformation simulations usually display a linear regime for small strain, even for single crystals with dislocations^27^. However, for larger strains, a non-linear elastic regime can precede dislocation activity due to the anharmonic interaction potential. Poly-crystalline simulations typically show non-linearities even at very small strain before dislocation activity starts due to grain boundary activity^28^.

The ultimate stress is reached at compressions of nearly 0.05, and the elastic limit is estimated to be a 0.02 strain for both nc-NTs and nc-NWs, on the basis of the loading and unloading plots. For all the cases studied, the NT and/or NW shapes do not significantly affect mechanical properties. This is because the grain boundary structure, in particular the ultrafine grains, seem to control several features, as the yield stress, the elastic limit, and the plastic behavior.

In addition to compressive loading we carried out the unloading of the systems from 0.1 and 0.2 strain. The stress-strain slope during recovery is similar to the loading slope. The residual strain is always larger than 0.07 and 0.17 for strains of 0.1 and 0.2, respectively. We observe that the crystalline nanotube and nanowire (Fig. 1a) have a larger residual strain than the nc-NTs and nc-NWs (Fig. 1b). Using the CAT tool^29^ it was observed that, for single crystals, more than 90% of defective atoms belong to twin boundaries, while only some isolated stacking faults remain after the unloading process. The large and non-recoverable twin boundaries, which often cross the whole diameter of the single crystal structure, are consistent with the larger residual strain observed for the single crystals as compared to polycrystals (Fig. S2). Unloading from a relatively small plastic strain does not lead to full de-twinning or recovery of the few existing dislocation junctions. In fact, at the end of the unloading process, the strain that corresponds to an initial 0.1 is 0.093 for c-NTs, 0.087 for c-NWs, and 0.077 for both nc-NWs and nc-NTs.

**Figure 2:**
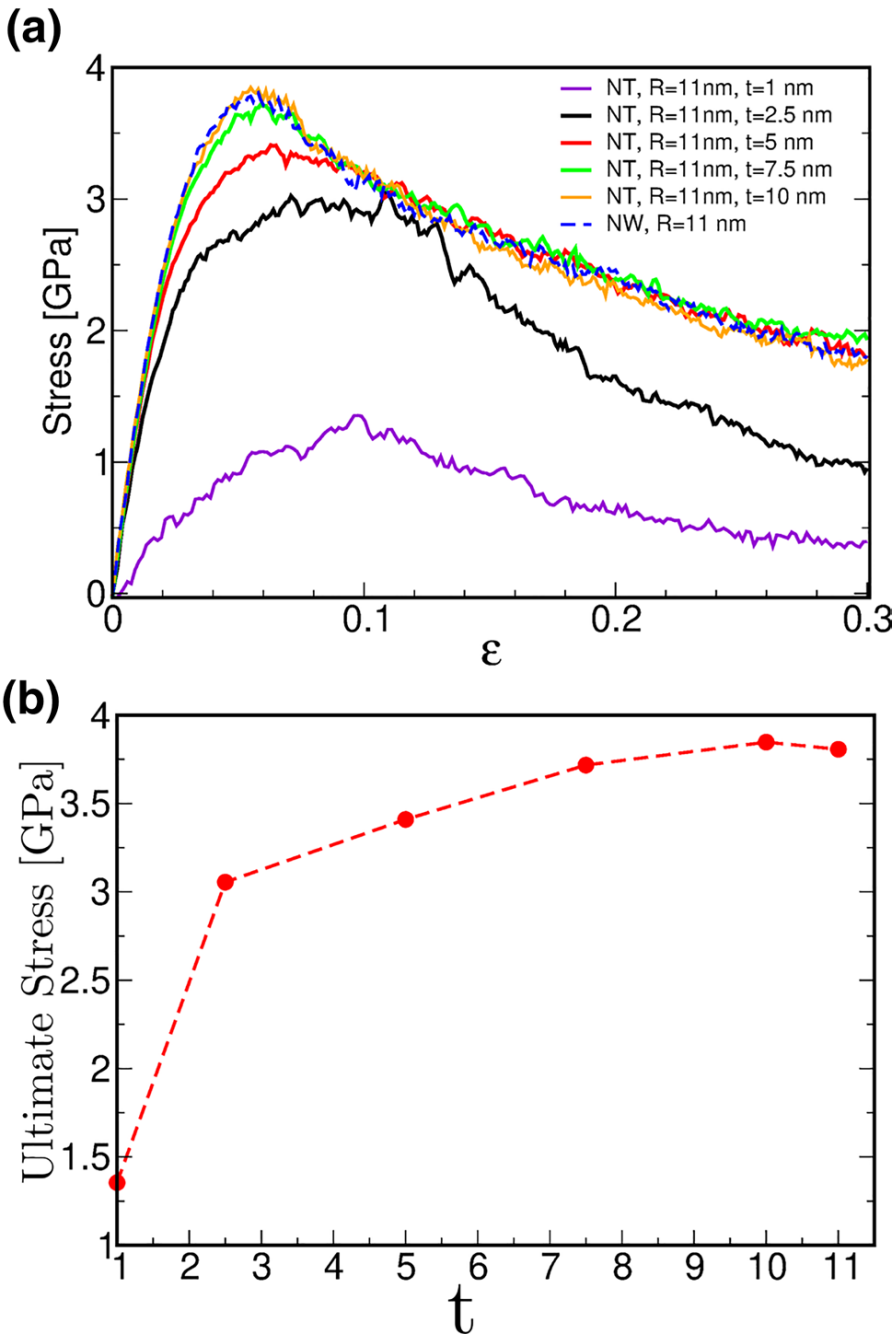
Mechanical response for different nc-NTs. (**a**) Compression stress-strain plots for different nc-NTs wall thicknesses (t). The blue dashed line corresponds to an 11 nm radius nc-Nw. (**b**) Ultimate stress as function of NT thickness.

nc-NTs and nc-NWs show similar mechanical performance. The nc-NTs wall thickness (t) dependence under compression is illustrated in Fig. 2a. As expected, the early plasticity stages are dominated by GB sliding; the thinner the thickness the earlier is the deviation from linear elastic behavior, and the lower the ultimate stress. The minimum shell thickness to obtain the same mechanical behavior than NWs is close to t = 7 nm, while values smaller than 5 nm have incidence not only on the maximum strength, but also on material plastic flow even for large deformations (Fig. 2b). For strains larger than 0.1 all curves are similar, except for the thinner thicknesses where the effective grain size is smaller, and surface effects are dominant, enhancing the grain boundary sliding towards the inner surfaces. We note that the hollow structure leads to mass reduction relative to the nanowire case. In Table 1, we summarize the relative mass with respect to nanowire mass for the cases we simulated, and the corresponding ultimate tensile stress, as displayed in Fig. 2b. We note that t = 5 nm represents a critical value, with a NW mass reduction of 30%, but which still has the same Young modulus and flow stress of the heavier NW, for strains larger than 0.1. In this case, the ultimate stress reduction is of only 0.4 GPa ($\epsilon$ = 0.1). For larger mass reduction, mechanical properties are not so well preserved.

**Table 1:** Ultimate stress, and relative mass ratio between nanotube ($m_{nt}$) and nanowire ($m_{nw}$), for different thickenesses. t = 11 nm represents the nanowire case.

t (nm)	1.0	2.5	5.0	7.5	10	11
%$\hbox {m}_{{nt}}$/$\hbox {m}_{{nw}}$	17.4%	40.3%	70.3%	89.9%	99.1%	100%
Ultimate stress (GPa)	1.35	3.05	3.41	3.71	3.84	3.80

**Figure 3:**
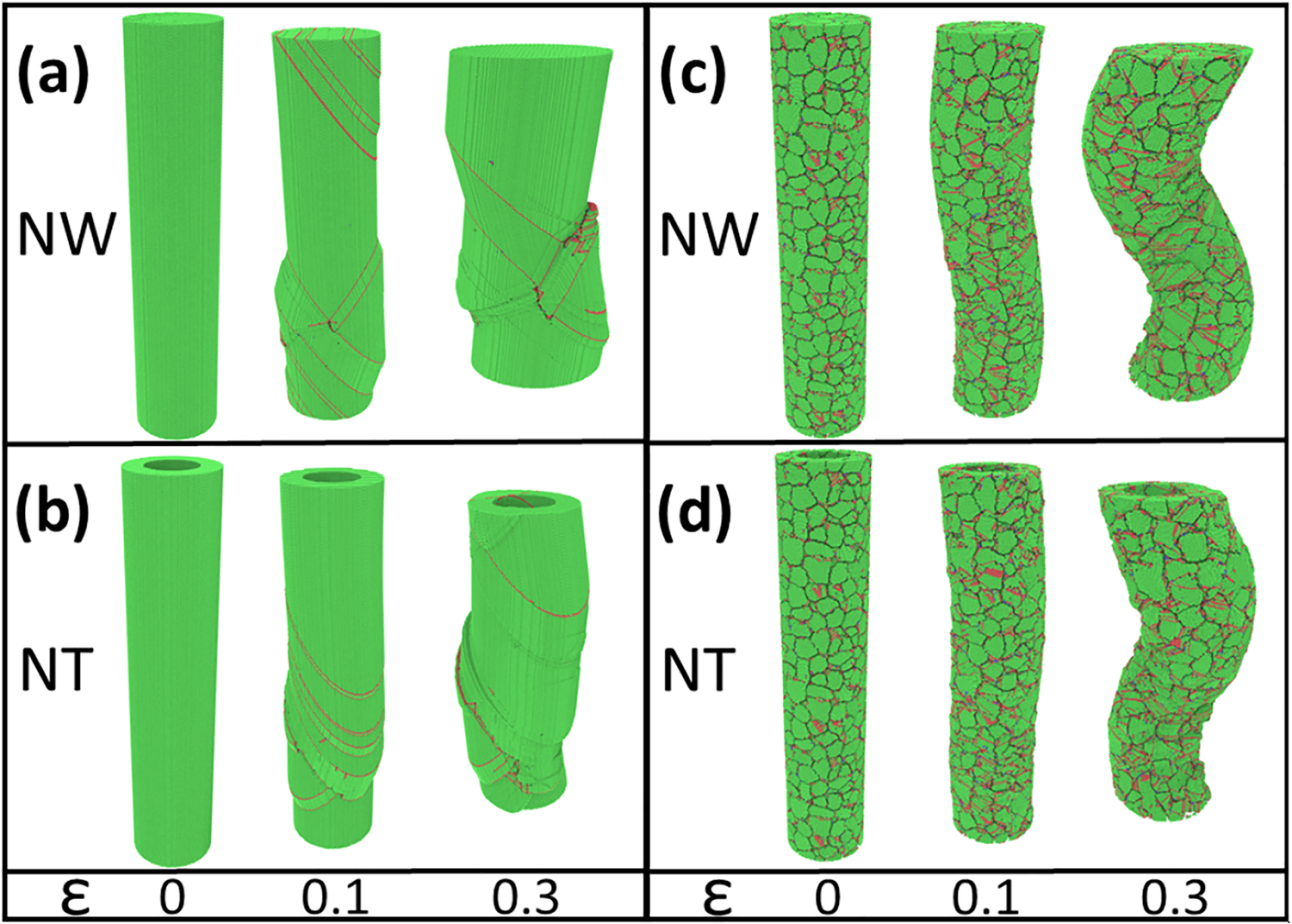
Illustration of NWs and NTs at different strains. (**a**,**b**) snapshots of crystalline NWs and NTs under different compression values. (**c**,**d**) nanocrystalline NWs and NTs under different compressions. The GB atoms are removed for clarity. The fcc (green), bcc (blue), and hcp (red) atoms were detected by common neighbor analysis (CNA).

As expected, in crystalline samples plasticity is dominated by partial dislocations along {111} planes, at $45^o$ to the loading axial direction, as illustrated in Fig. 3a,b. Several stacking faults, located in consecutive planes, lead to twinning. Stacking faults and twins lead to shear localization, and a noticeable bulging is appreciated for strains larger than 0.1. However, in nanocrystalline systems the situation is quite different, since the small grain size ($<5$ nm) implies that the material can be assumed to be an almost isotropic solid, where dislocation activity also takes place in the {111} planes, but with many orientations and dislocations of a characteristic length not larger than the grain size, leading to a buckling deformation mode, instead of the bulging observed in single crystals (Fig. 3c,d). It is known that in some NWs under compression dislocation activity can dominate plasticity^23,30,31^; however, grain boundary sliding cannot be completely ruled out. Figures 3c,d, show that strains of 0.1 lead to an increase in roughness, with a $\Delta r$ value of 0.8 nm along nc-NT and nc-NW surfaces. For larger strains ($>0.20$) grain boundary activity leads to buckling, with $\Delta r \ge 3.5$ nm for both nc-NTs and nc-NWs.

In order to follow the grain evolution of nanocrystalline NTs and NWs we used the local crystalline orientation^32^ depicted in Figs. S3, and S4, respectively. At low strains, both NTs and NWs show grains with few defects and almost no rotation. However, for strains larger than 0.1 the coiling shows clear differences, while the buckling wavelength of the NTs and NWs remains almost constant. Thus, we notice that there are still some discrepancies about the validity of continuum buckling theory at the nanoscale^33^. However, if we assume buckling could be described by the Euler-beam theory, then it will be affected by the aspect ratio of the nanotubes^34^.

**Figure 4:**
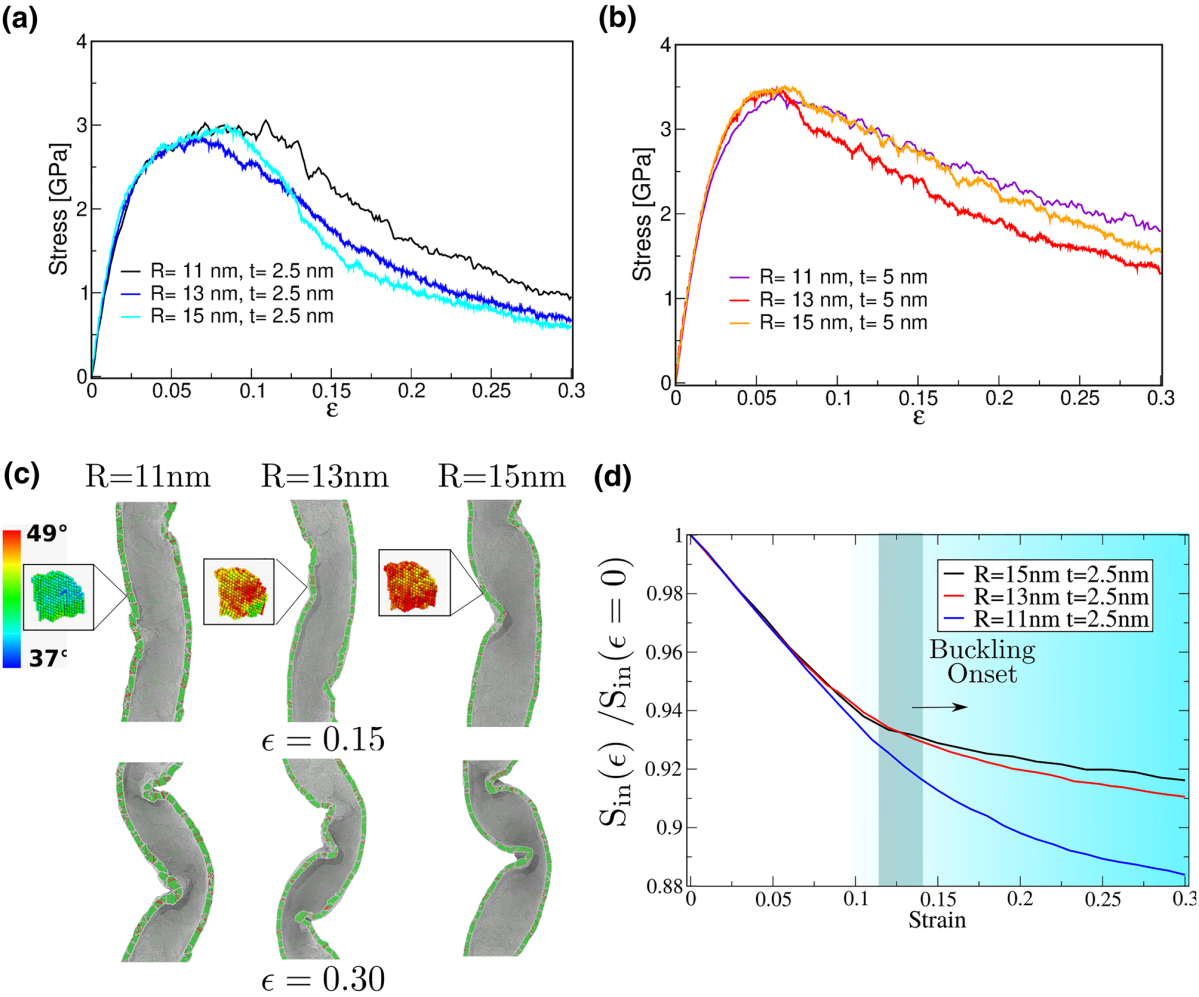
Role of NT diameter on the mechanical response of nc-NTs. (**a**) Stress-strain curve for nc-NTs of R = 11, 13, and 15 nm and *t* = 2.5 nm. (**b**) Stress strain curve for nc-NTs of R = 11, 13, and 15 nm, and *t* = 5 nm. (**c**) nc-NT buckling and coiling. Atoms are colored according to the crystalline structure obtained with a CNA analysis: green = fcc, red = hcp, and white = other. Insets show the rotation angle of a single grain, obtained from the Polyhedral Template Matching algorithm (PTM), relative to zero strain. (**d**) depicts the inner surface ($S_{in}$) fraction vs. strain, where the darker gray region delimits the buckling onset.

To investigate the role of the radius we simulate R = 13 and 15 nm NTs for t = 2.5 nm and 5 nm. As is observed in Fig. 4a,b the NT thickness is the most relevant parameter to describe the mechanical behavior. For both radius, the maximum stress is achieved for 5 nm, with a similar flow stress at larger strains, while the weaker response is obtained from t = 2.5 nm. As it was previously mentioned, the ultra-small wall enhances material migration to the inner surface. The consequence of grain activity, in particular, grain boundary rotation, appears as surface buckling during the coiling process, as seen in the inset of Fig. 4c.

Buckling appears above 0.15 strain and contributes to plasticity. We investigated the tube geometry, shown in Fig. 4c, and find that the inner diameter of the tube reaches values as small as $\sim$2 nm at the buckling site, which is a reduction of nearly 90% of the initial diameter. To study in detail this geometry we computed the surface and volume variations of the NT inner surface. The volume inside the tube decreases linearly with strain, with a slope that is almost unchanged by buckling, as shown in Fig. S5. This is because buckling is highly localized and does not significantly affect the total pore volume. The inner surface of the NT shows this localization in Fig. S6. Inner surface area is expected to decrease due to uniaxial deformation, but increase due to buckling. The net effect, shown in Fig. 4d, is that the inner surface area continues to decrease after buckling, but with a significantly smaller slope.

### Dislocation analysis of Ni NTs and NWs

**Figure 5:**
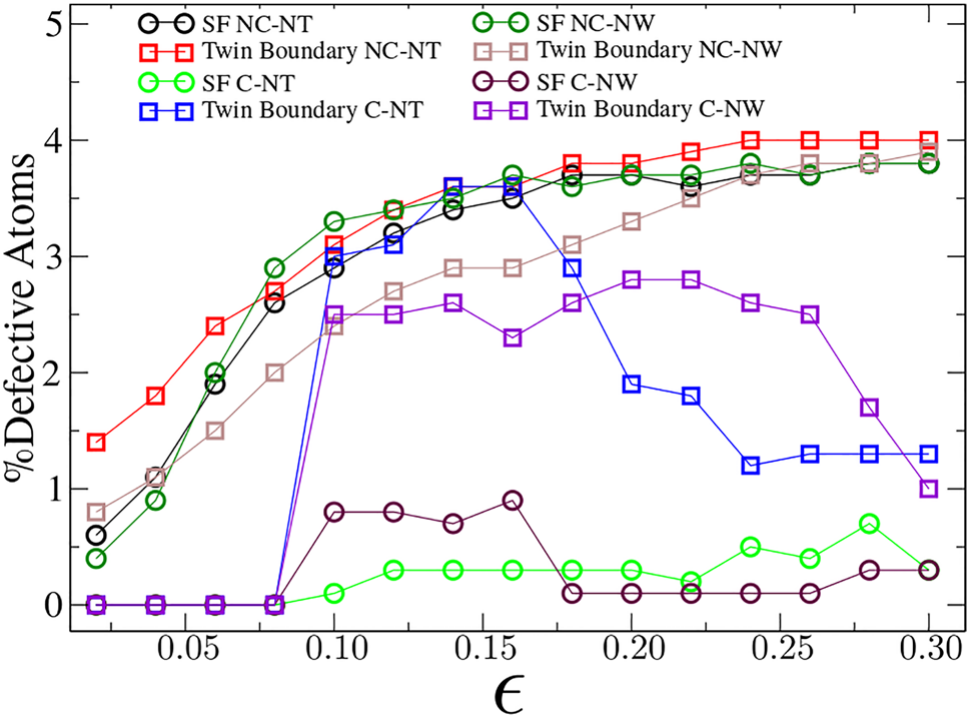
Planar defect analysis for NTs of t = 5 nm and R = 11 nm, and NWs of R = 11 nm. Defects were calculated with CAT, identifying the percentage of defective atoms in SFs and twin boundaries, for different strains.

Figure 5 illustrates major differences in planar defect formation of crystalline and nc structures, and their evolution. In c-NTs and c-NWs a compressive strain of approximately 0.07 is required for nucleation of the earliest planar defects. In both cases plasticity is mostly dominated by twinning (there should be a narrow strain range where stacking faults (SFs) dominate before they lead to twins). In nc systems SFs and twins already appear at zero strain since the ultra-small grain size allows the nucleation of faults during nc relaxation. Major differences arise for crystals since both structures, NTs and NWs, seem to be prone to create more planar defects as a function of applied strain. Moreover, the nc-NT and nc-NW geometries have the same behavior, even at strains close to 0.3; thus, we conclude that the formation of planar defects is dominated by the grain boundary structure and not by the tubular or wire design. In nc-NTs under tension, Rojas-Nunez revealed twin dominated fracture^17^; however, under compression a different deformation mechanism is observed, at least for nc systems where bending can replace the shear localization observed in the crystalline case. Here nc-NTs and nc-NWs present more SFs and twins in the concave portions of the tube, where the curvature is more relevant, than in the convex regions.

**Figure 6:**
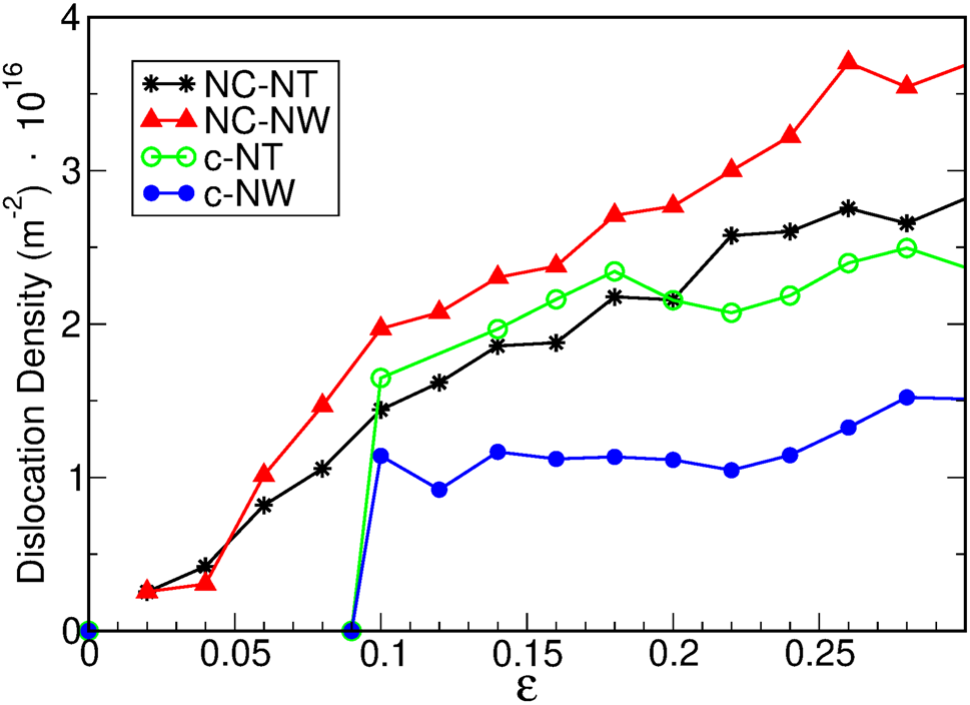
Dislocation density of t = 5 nm and R = 11 nm Ni NTs, and R = 11 nm NWs. Both crystalline and nc structures are compared.

Dislocation analysis, illustrated in Fig. 6, shows that some dislocations appear at strains less than 0.02, from GB sources. Plastic strain due to dislocations is limited by the fact that dislocation lines are typically not longer than the grain diameter, and only move across a single grain. Therefore, there is a scenario of dynamical absorption and production of dislocations taking place at grain boundaries.

**Figure 7:**
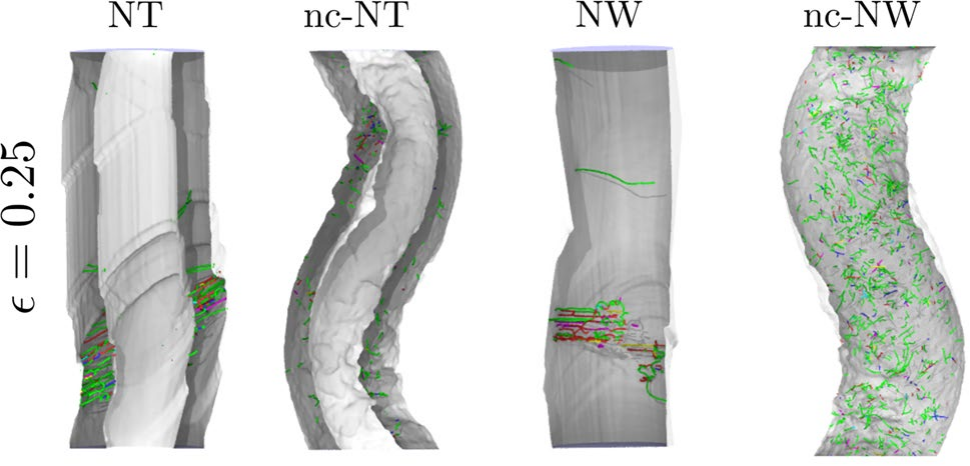
Dislocations for a NTs with t = 5 nm and R = 11 nm NTs, and for NWs of R = 11 nm NWs compressed to a strain of 0.25. Only dislocations and surfaces are illustrated in the figure. We notice that, due to the presence of two surfaces, several NT dislocations are not apparent.

Dislocation counts in nc-NTs and nc-NWs show that Shockley partials (1/6<112>) dislocations correspond to 90% of the total count, while 5% are Stair-Rod (1/6<110>) junctions. The rest corresponds to small segments of full dislocations (1/2<110>). In all the cases illustrated, single-crystal systems show a slower growth of dislocation density with strain, after plastic yielding. Dislocations can grow and move across the whole cross-section of the wire or NT, not only across a single grain. There is evidence of high localization of Hirth and Stair-Rod dislocations, originating from dislocation junctions, in regions confined by twin boundaries and SFs, as seen in Fig. 7. This differs from nc-systems, where dislocations tend to be distributed over the whole NT.

## Conclusions

The main goal of our study of nc-NTs is motivated by its adjustable combination of geometrical parameters that mimic NW performance, but are significantly lighter and can adopt functional shapes. In this work we study, by means of molecular dynamics simulations, the uniaxial compressive response of NTs and NWs, with and without grain boundaries. We note that the inclusion of the grain boundary structure is a more realistic approach in the modeling of metallic NTs, as the ones obtained experimentally^15–17^.

Our conclusions can be summarized as follows:

- For the grain sizes studied here a NT wall thickness larger than a few nm is necessary to achieve the same mechanical properties than the solid NW counterpart. Smaller thickness are dominated by surface effects and an enhanced grain boundary activity, resulting in softer structures than their thicker counterparts.
- In crystalline NTs and NWs, plasticity is always dominated by twinning and by stacking faults, led by Shockley partial dislocations in highly localized regions. In nc counterparts, GB activity also contributes significantly to plasticity, but the small grain size of the nc material leads to a uniform dislocation distribution over the whole nanostructure, avoiding any possible shear localization as observed in single crystal structures.
- The absence of only a few preferential planes allows a completely flexible behavior for nc-structures at larger strains. Crystalline shear localization is substituted by nc coiling for strains even of 0.30. In addition, for t = 2.5 nm, plastic deformation is dominated by the NT and wall thickness buckling, both taken place simultaneously. The surface buckling is activated by the small thickness, which leads to material diffusion to the NT inner surface.
- The inner volume of the nanotubes, which could be used for transport or storage, is reduced by compression and plastic activity. Buckling can affect the rate at which this reduction occurs, and also lead to significant localized constrictions of the inner cavity. Inner surface area is also reduced by compression, and buckling diminishes its reduction rate with strain.
- Substantial differences in the mechanical properties of crystalline and nanocrystalline systems have been observed. Plastic deformation starts at larger strain for perfect crystals, due to the absence of pre-existing defects in the material^35^. In our case, dislocations nucleate at the surface of the c-NTs or c-NWs, as expected. On the other hand, grain boundary activity in polycrystalline systems can produce plastic deformation at small strains. At larger strains dislocations can nucleate from surfaces or from grain boundaries^36,37^.
- In the case of c-NWs and c-NTs, plasticity starts with surface nucleation of Shockley partial dislocations, which travel across the NT and lead to surface steps. They produce stress drops, generating a seesaw stress-strain curve, up to a strain of 0.1. Dislocation nucleation in single crystal “perfect” NWs and NTs requires larger stress than GB activity, leading to a higher elastic limit and ultimate stress than for nc-NWs and nc-NTs. The abundance of partial dislocations leads to twinning, which also reflects on surface steps and bulging in regions of high dislocation density.
- nc-NTs and nc-NWs display a different plastic behavior, without bulging. This is because a significant amount of strain is accommodated by GBs, and dislocation activity is confined by GBs which can stop or absorb impinging dislocations. The large number of grains in random orientations leads to a large number of dislocation glide-plane orientations and, therefore, the material responds as a nearly isotropic material. In this case, grain rotation in the material under high compression allows for buckling, which depends on the NT geometrical parameters, such as thickness, radius, and NT length.

Our findings are useful for the design of new light-weight and highly deformable meta-materials, taking advantage of the mass reduction and the ductile behavior of nc-NTs. Moreover, these nc-structures are softer than their defect-free crystalline counterparts. Future work will focus on grain size effects and grain boundary engineering, as possible mechanisms to modify mechanical properties.

## Methods

### Computational modeling of Ni nanostructures

The uniaxial compressive strain is simulated by molecular dynamics employing the LAMMPS code^38^. The Ni interatomic interactions are modeled using the embedded atom method^39^, with the parametrization proposed by Mishin et al.^40^, that correctly reproduces several physical quantities such as elastic constants^41,42^, and stacking fault energy^43^, amongst other properties. The nanocrystalline texture was obtained by cutting NWs and NTs from a Ni nc-bulk sample of 5 nm mean grain size. The nanocrystal bulk with specified grain sizes was built employing a Voronoi tessellation algorithm^45^, which provides a grain size distribution similar to the one of experiments that use atomic layer deposition techniques^15^.

Cuts of the bulk sample lead to a decrease in mean grain size. To estimate the new mean grain size in NWs and NTs, grains were identified by removing GB atoms with the help of Common Neighbor Analysis (CNA), and next using cluster analysis to isolate each grain as a given cluster. Atoms in the cluster can be separated between “surface” and “interior” atoms using centrosymmetry parameter analysis. For each cluster, a center of mass (COM) is calculated, and the distance between that COM and the atoms at the periphery of the cluster gives a distribution of radii, leading to a mean grain radius. This is the same method used in the study of Ni NTs under tension^17^. As a check for the method, when applied to the bulk nc sample, we verified that it gives 5  nm grain size. For NTs with thickness thinner than the mean grain size, grains can have a length, along the NT axis, larger than the NT thickness; this can yield a mean grain size larger than the thickness itself. This is indeed the case for t = 2.5 nm, where we find a mean grain size of 3 nm. Something similar has been found for hollow nanocrystalline spheres^44^, where the interplay between thickness and the original grain size allows for tailoring of the mechanical properties. Future studies for nanotubes might also explore this interplay.

Voronoi constructions yield grain boundary structures far from equilibrium; therefore, high energy grain boundaries have to be relaxed to avoid possible artifacts. The grain boundary structure was relaxed by means of a zero-pressure barostat coupled to a 300 K Nose-Hoover thermostat; additional details of this methodology can be found elsewhere^45–48^. Finally, NWs and NTs of the desired radius and thickness are carved from the nc template and relaxed again, with a velocity rescaling algorithm, at 300 K during 100 ps, coupled to a zero-pressure barostat in the axial direction. This cut slightly modifies the grain size distribution, and the resulting mean grain sizes are 3.8 nm and 3.5 nm for NWs and NTs, respectively. Simulations were performed using periodic boundary conditions along the nanotube axis (*z*-axis).

The 11, 13 and 15 nm radius NTs, of 2.5 and 5.0 nm thickness, were created to investigate the role played by the NT radius. Additionally, for the smaller NTs, the wall thickness t was varied (2.5, 5.0, 7.5 and 10nm), to investigate the role of thickness in plasticity. A minimum shell thickness of 2.5 nm was adopted to avoid partial collapse and to ensure a uniform nanotube, before uniaxial compression was performed. This criterion was chosen based on Cao et al.^21^ findings, which show a strong surface reorientation when the NT thickness is close to 1 nm. We note that this behavior has also been observed in other hollow structures^49–51^, for a specific combination of radius and thickness. In those cases, the surface stress is not compensated by the shell, originating a partial shrinkage, and the surface reorientation is assisted by Shockley partial dislocations as the dominant mechanism. As a consequence, the hollow structure reduces its radius, or roughens its surface to decrease surfaces stress.

### Mechanical deformation and molecular dynamics

Uniaxial compression was applied along the z-axis at a strain rate of $10^8 s^{-1}$, and with a time step of 1.0 fs; the same rate was applied when unloading to zero stress. During deformation, periodic boundary condition in the *z*-axis were adopted. The system temperature was held at 300 K during the simulations, by means of a Nose-Hoover thermostat. The NTs and NWs were uniaxially deformed along *z* using an NPT ensemble to keep zero pressure in *x* and *y*. Each nanostructure was then homogeneously compressed to deformations of 30%. Defect detection, visualization, and rendering were carried out with OVITO^52^ (https://www.ovito.org), while dislocations and planar defect identification were obtained with the Dislocation Extraction Algorithm (DXA) developed by Stukowski^52^, and the Crystal analysis Tool^29^ (CAT) (https://gitlab.com/stuko/crystal-analysis-tool).

As a measure of roughness, we computed the standard deviation ($\Delta r$) with respect to the average NT radius, as

$$\begin{aligned} \Delta r= \sqrt{\sum _{i=1}^n\frac{(r_i-\langle r \rangle )^2}{n}}\,, \end{aligned}$$

where $r_i=\sqrt{x_i^2+y_i^2}$, and $\langle r\rangle =\frac{1}{n}\sum _{i=1}^nr_i$ is the average NT radius.

## Supplementary information

Supplementary Information.
