# Supplementary material for: Polycrystalline Ni nanotubes under compression: a molecular dynamics study
Source: Sci Rep. 2020 Dec 3;10:21096. doi: 10.1038/s41598-020-76276-y (PMC7713178; doi:10.1038/s41598-020-76276-y)
Supplement: Supplementary file 1 — Supplementary Information. [file 41598_2020_76276_MOESM1_ESM.pdf]

Supporting Information
Polycrystalline Ni Nanotubes under Compression: A Molecular Dynamics Study

J. Rojas-Nunez[1,2], S. E. Baltazar[1,2], R. I. Gonzalez[2,3], E. M. Bringa[3,4], S. Allende[1,2], M. Kiwi[2,6], F. J. Valencia[2,5,*]

[1]Departamento de Física, Universidad de Santiago de Chile, USACH, Av. Ecuador 3493, Santiago, Chile.
[2]CEDENNA, Universidad de Santiago de Chile, USACH, Av. Ecuador 3493, Santiago, Chile.
[3]Centro de Nanotecnología Aplicada, Facultad de Ciencias, Universidad Mayor, Chile
[4]CONICET & Facultad de Ingeniería, Universidad de Mendoza, Mendoza 5500, Argentina
[5]DAiTA Lab, Facultad de Estudios Interdisciplinarios, Universidad Mayor, Santiago, Chile.
[6]Departamento de Física, Facultad de Ciencia, Universidad de Chile, Chile.

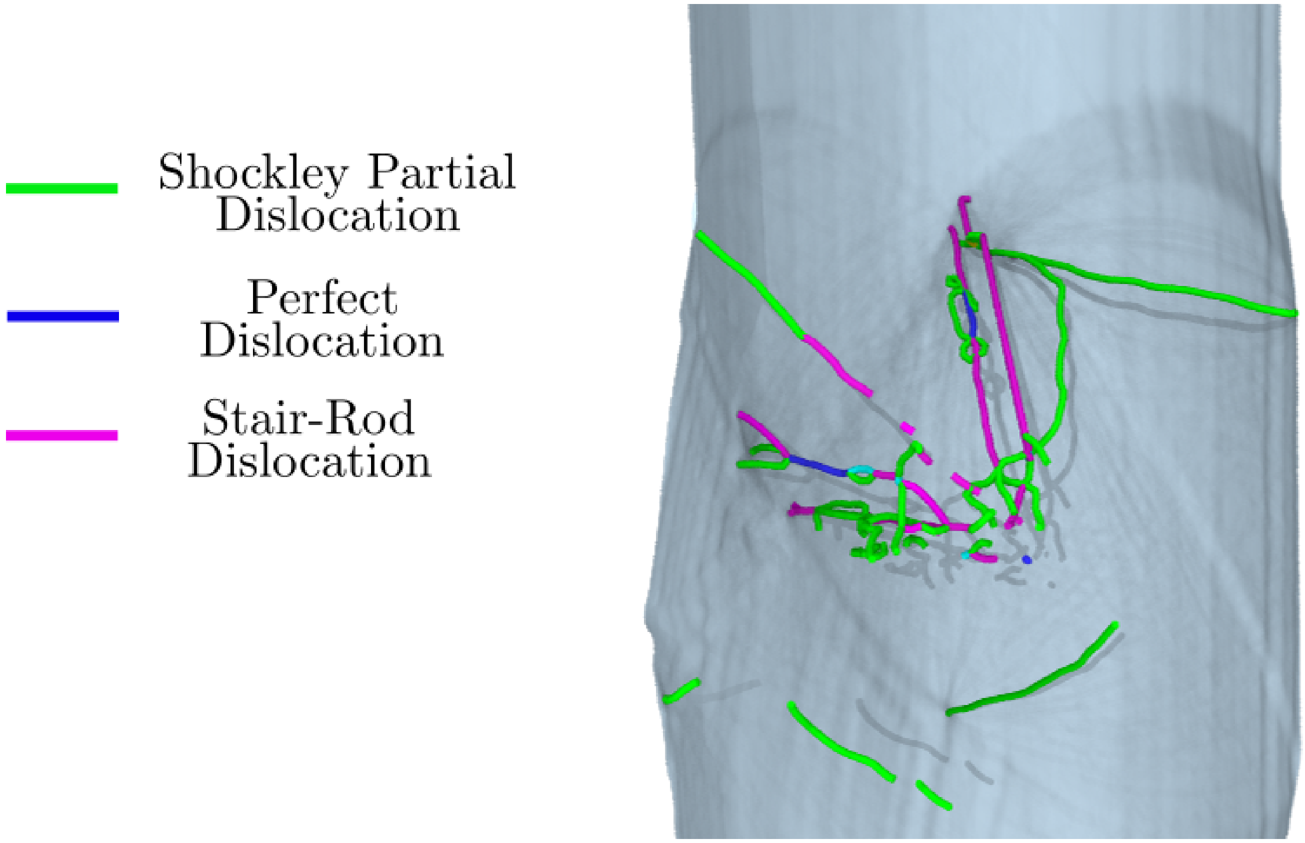

Figure S1. Dislocation network in the c-NW at $\varepsilon = 0.1$. Dislocation analysis shows that dislocations are mostly Stair-Rod (25%), and Shockley partial dislocations(69%). The grey surface delimits the NW volume. Atoms were deleted from figure to show only the dislocation network. Rendering was performed with OVITO[1]

References

1. Stukowski, A. Visualization and analysis of atomistic simulation data with ovito–the open visualization tool. *Model. Simul. Mater. Sci. Eng.* **18**, 015012, DOI: 10.1088/0965-0393/18/1/015012 (2010).

2. Stukowski, A. Structure identification methods for atomistic simulations of crystalline materials. *Model. Simul. Mater. Sci. Eng.* **20**, 045021, DOI: 10.1088/0965-0393/20/4/045021 (2012).

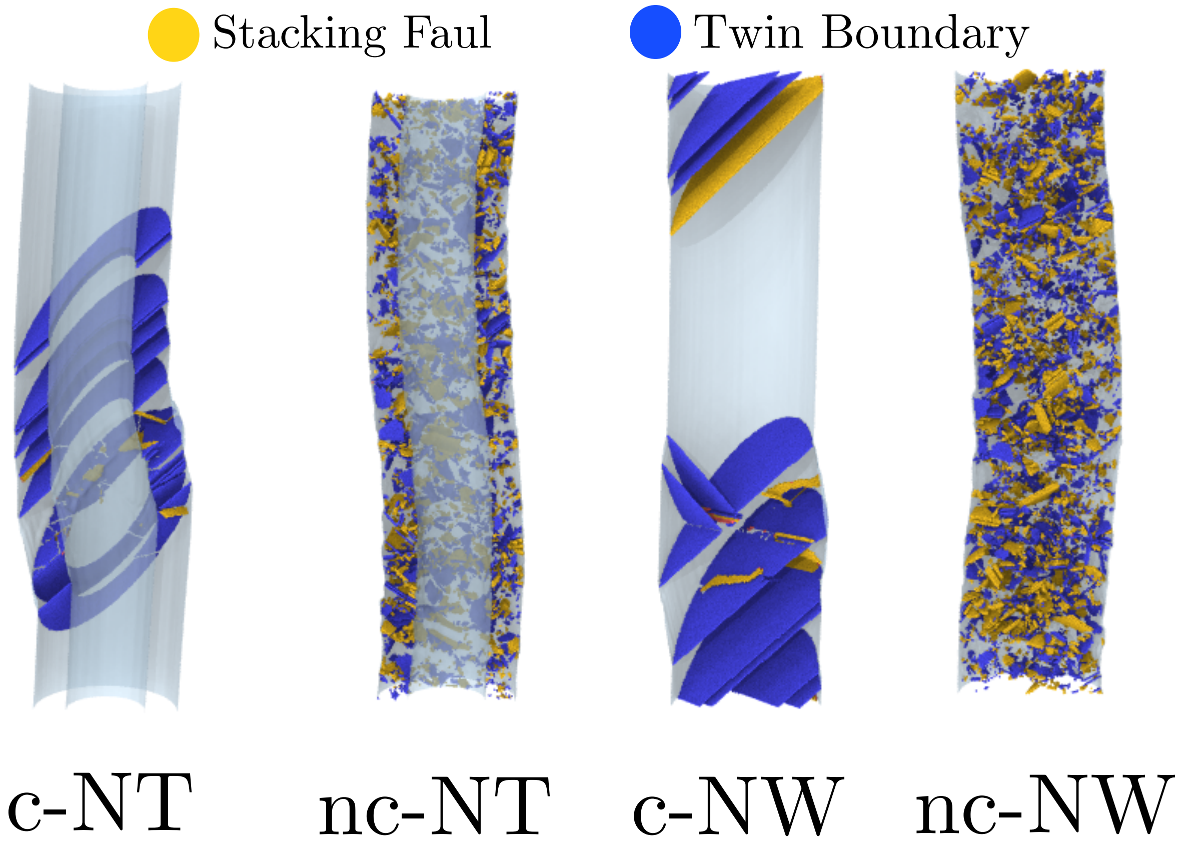

Figure S2. Snapshots of NT and NW after unloaded compression, showing the stacking Faults (orange), and Twin Boundaries (blue) detected with the Crystal Analysis Tool[2]. For illustrative purpouses fcc atoms were deleted from figure. Shaded region delimits the inner and/or outer surface of the NT and NW. Rendering was performed with the OVITO code[1]

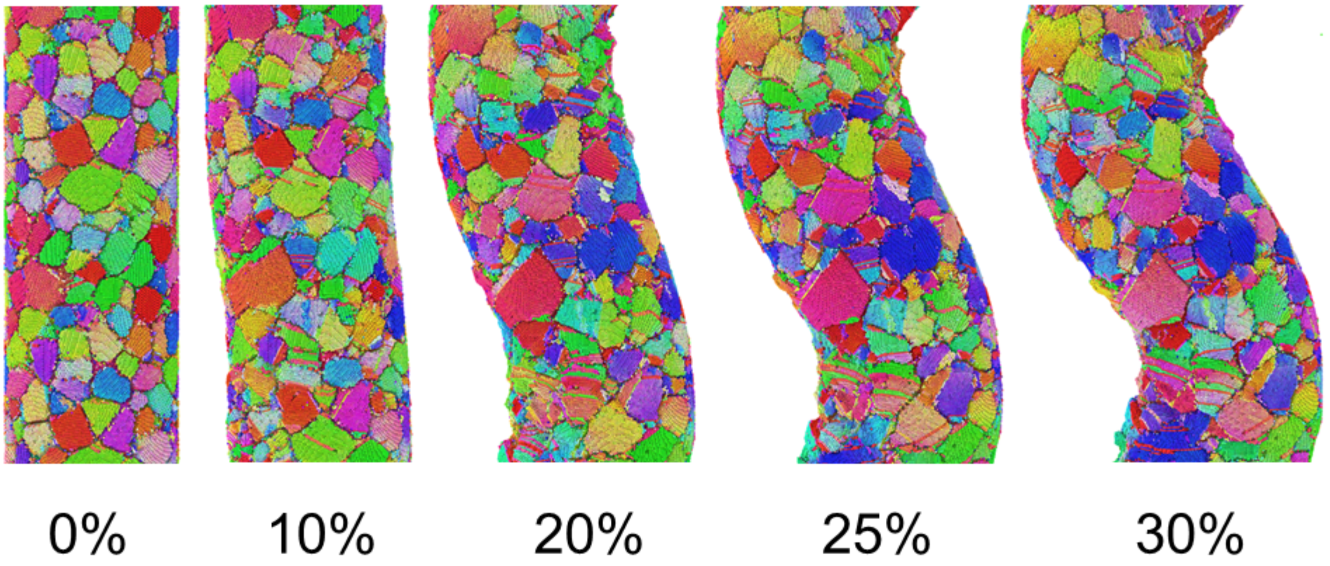

Figure S3. Local crystallographic orientation of Ni NT with thk=5nm under compression. Grains are depicted and colored to visualize crystallographic orientation (red=[001], green=[011], blue=[111]).Rendering was performed with the OVITO code[1]

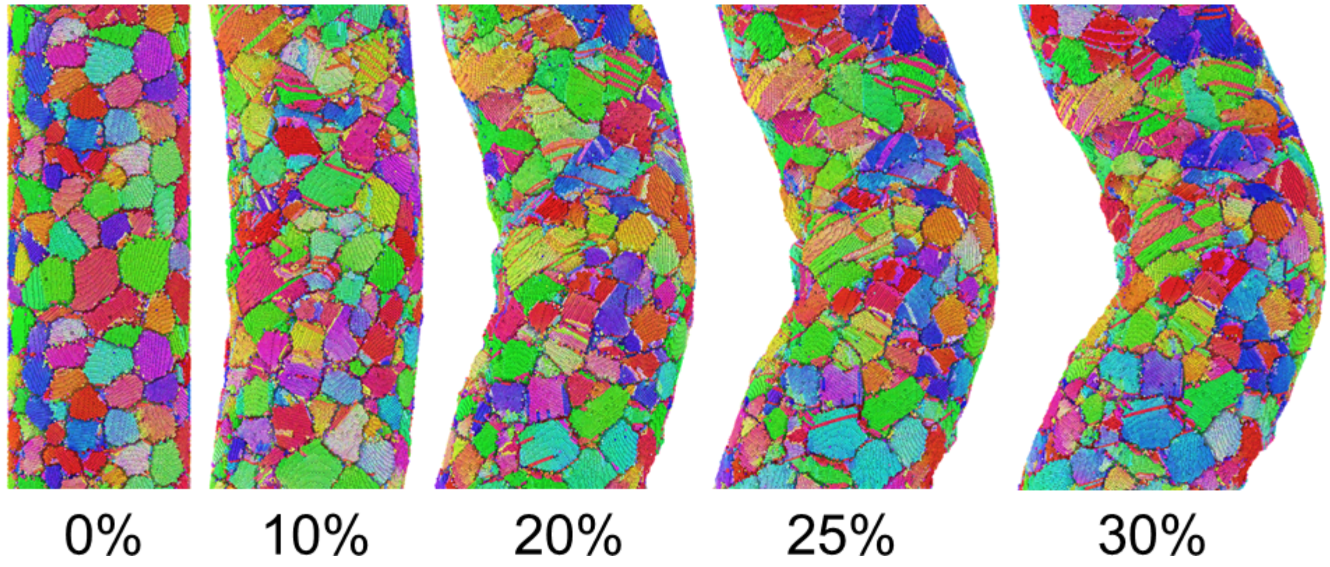

Figure S4. Local crystallographic orientation of Ni NW under compression. Grains are depicted and colored to visualize crystallographic orientation (red=[001], green=[011], blue=[111].Rendering was performed with OVITO.[1]

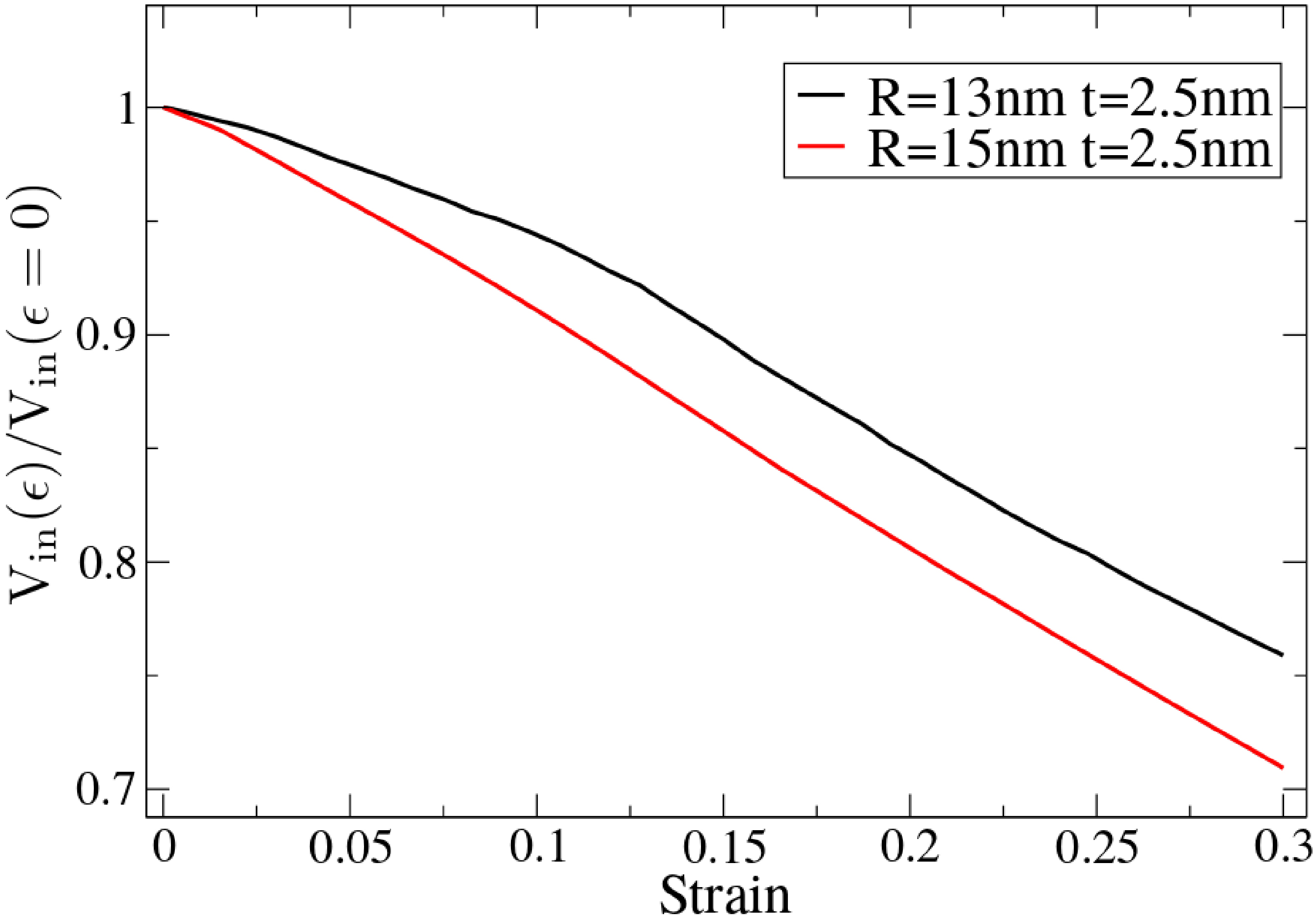

Figure S5. Pore volume (V_{in}) as strain function. Results are normalized with respect to the initial pore volume.

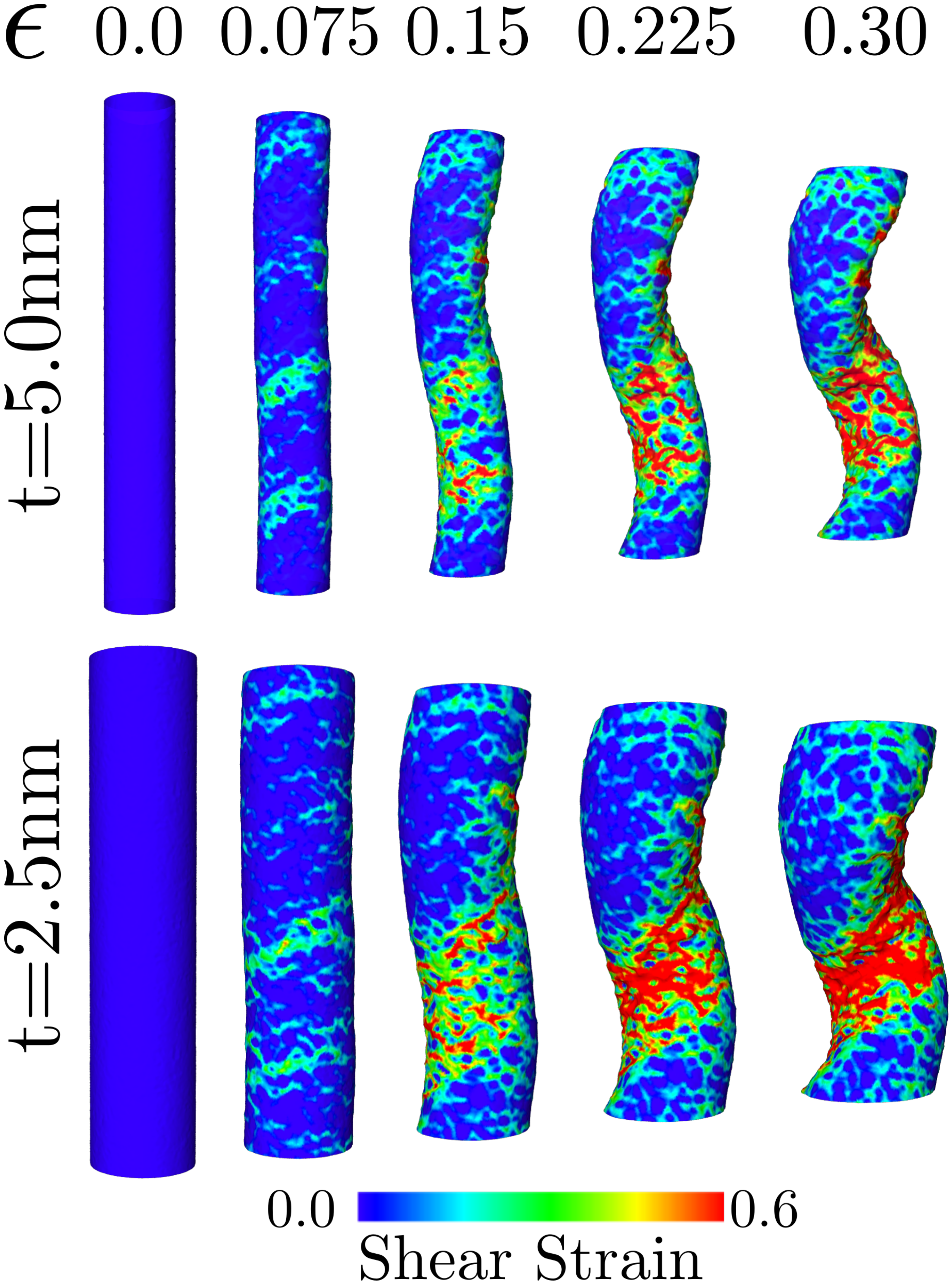

Figure S6. Shear strain on NT inner surface. Both cases illustrate a NT of R=15nm, and t=2.5nm and t=5nm.Rendering was performed with OVITO.[1]
